# Efficacy of a Comprehensive Weight Reduction Intervention in Male Adolescents With Different FTO Genotypes

**DOI:** 10.1002/edm2.483

**Published:** 2024-03-31

**Authors:** Zahra Roumi, Zahra Salimi, Zahra Mahmoudi, Khadijeh Abbasi Mobarakeh, Maryam Ladaninezhad, Mobina Zeinalabedini, Mohammad Keshavarz Mohammadian, Ali Shamsi‐Goushki, Zahra Saeedirad, Bojlul Bahar, Sara Khoshdooz, Naser Kalantari, Ghasem Azizi Tabesh, Saeid Doaei, Maryam Gholamalizadeh

**Affiliations:** ^1^ Department of Nutrition, Science and Research Branch Islamic Azad University Tehran Iran; ^2^ Nutrition and Metabolic Diseases Research Center Ahvaz Jundishapur University of Medical Sciences Ahvaz Iran; ^3^ Food Security Research Center and Department of Community Nutrition, School of Nutrition and Food Science Isfahan University of Medical Sciences Isfahan Iran; ^4^ School of Nutritional Sciences and Dietetics Tehran University of Medical Sciences Tehran Iran; ^5^ Department of Cellular and Molecular Nutrition, School of Nutritional Sciences and Dietetics Tehran University of Medical Sciences Tehran Iran; ^6^ Department of Nutrition, School of Medicine Mashhad University of Medical Sciences Mashhad Iran; ^7^ Department of Clinical Nutrition and Dietetics Tehran University of Medical Sciences Tehran Iran; ^8^ Nutrition Sciences and Applied Food Safety Studies, Research Centre for Global Development, School of Sport & Health Sciences University of Central Lancashire Preston UK; ^9^ Faculty of Medicine Guilan University of Medical Science Rasht Iran; ^10^ Department of Community Nutrition, Faculty of Nutrition and Food Technology National Nutrition and Food Technology Research Institute, Shahid Beheshti University of Medical Sciences Tehran Iran; ^11^ Genomic Research Center Shahid Beheshti University of Medical Sciences Tehran Iran; ^12^ Cancer Research Center Shahid Beheshti University of Medical Sciences Tehran Iran

**Keywords:** BMI, *FTO*, gene polymorphism, obesity

## Abstract

**Background:**

The *FTO* gene polymorphisms may influence the effects of lifestyle interventions on obesity. The present study aimed to assess the influence of the rs9930506 *FTO* gene polymorphism on the success of a comprehensive weight loss intervention in male adolescents with overweight and obesity.

**Methods:**

This study was carried out on 96 adolescent boys with overweight and obesity who were randomly assigned to the intervention (*n* = 53) and control (*n* = 43) groups. The blood samples of the participants were collected, and the *FTO* gene was genotyped for the rs9930506 polymorphism. A comprehensive lifestyle intervention including changes in diet and physical activity was performed for 8 weeks in the intervention group.

**Results:**

Following the lifestyle intervention, BMI and fat mass decreased significantly in the intervention group compared with the control group (both *p* < 0.05), while no change was found in weight, height or body muscle percentage between the groups. The participants in the intervention group with the AA/AG genotype and not in carriers of the GG genotype had a significantly higher reduction in BMI (−1.21 vs. 1.87 kg/m^2^, *F* = 4.07, *p* < 0.05) compared with the control group.

**Conclusion:**

The intervention in individuals with the AA/AG genotype has been significantly effective in weight loss compared with the control group. The intervention had no association effect on anthropometric indices in adolescents with the GG genotype of the *FTO* rs9930506 polymorphism.

**Trial Registration:**

Name of the registry: National Nutrition and Food Technology Research Institute; Trial registration number: IRCT2016020925699N2; Date of registration: 24/04/2016; URL of trial registry record: https://www.irct.ir/trial/21447

## Graphical abstract

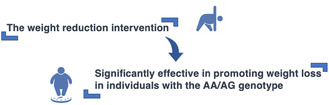

## 1 Introduction

Overweight and obesity in children and adolescents are frequently reported to be serious health problems in developing countries [1]. Between 2000 and 2016 in Iran, the prevalence of obesity rose by 5.4% among boys and 4.2% among girls [2]. Additionally, the rate of overweight among Iranian students was higher in boys at 14% than at 8% in girls [3, 4]. Overweight and obesity affect several aspects of health and well‐being in children and adolescents and increase the risk of developing diabetes, hypertension, dyslipidaemia and depression in adulthood [5].

Obesity is a multifactorial and complex health issue. Genetics and lifestyle factors including the quality and quantity of dietary intake and the extent of physical inactivity play key roles in the aetiology of obesity [6, 7]. In recent years, the increase in the portion size of food and getting more calories by consuming fast foods and high‐calorie sweet drinks have been the main dietary changes that have led to weight gain in children and adolescents [8], although most approaches to weight management have promising results and often provide a short‐term effect with regaining weight at a later stage.

Recent studies have demonstrated that the interaction between genetics and environmental factors plays an important role in overweight and obesity [9]. Some genetic variations may influence the association between obesity and lifestyle through different mechanisms. For example, a number of single nucleotide polymorphisms (SNPs) in the fat mass and obesity (*FTO*) gene such as rs9939609, rs9930506, rs6499640, rs8050136 and rs1558902 were reported to be associated with obesity in both children and adults [10, 11, 12]. The *FTO* gene regulates the synthesis of peptides involved in food intake and thereby may influence the energy haemostasis of the body [13]. A study of genetic association indicated that the SNP rs9930506 of the *FTO* gene has a strong association with obesity indices such as weight and hip circumference [14].

Moreover, the role of the *FTO* gene polymorphism in the effectiveness of weight loss interventions is recently investigated. A recent meta‐analysis reported that individuals carrying obesity‐predisposing allele (AA or AT genotype) in rs9939609 had a greater weight loss caused by dietary and lifestyle interventions than those with the TT genotype [15]. However, most studies are conducted on adults, and the results on the interactions of *FTO* polymorphisms with lifestyle factors and body weight management are contradictory. For instance, one study found that *FTO* rs9930501, rs9930506 and rs9932754 were not associated with any post‐intervention differences in dietary intake, anthropometric and cardio‐metabolic parameters after 6 months of dietary intervention [16]. Furthermore, the effect of weight reduction interventions on adolescents with different *FTO* genotypes is not clear. So, the aim of this study was to investigate the effect of rs9930506 polymorphism on the success of weight reduction interventions in male adolescents with overweight and obesity.

## 2 Methods

### 2.1 Participants

This field trial study was carried out on male students with overweight and obesity, aged between 12 and 16 years who were selected by simple random sampling from two schools in District 5 of Tehran, Iran. Due to the gender segregation of schools in Iran and the fact that the prevalence of obesity in male adolescents is higher than that of female adolescents (13.58 vs. 10.15%, respectively) [3], this study was conducted on male students. Two schools with considerable homogeneity in terms of the social and economic backgrounds of the students were randomly assigned as the intervention school and the control school. The sample size was estimated using an online tool (http://www.openepi.com/) and considering *α* = 0.95, *β* = 20% and power of 0.8. Only students who expressed a willingness to participate and had obtained written consent from both themselves and their parents or guardians were considered. Other inclusion criteria were body mass index (BMI) exceeded the 1+ standard deviation (*z*‐score) from the norm for their respective age and sex, and maturity of the participants. Maturity was gauged through physical benchmarks linked to the Tanner stages, and boys with grip strength above 25 kg and height over 65 inches were classified as mature. On the exclusion side, the study meticulously excluded individuals who were diagnosed with medical conditions known to influence body weight included uncontrolled chronic diseases affecting weight, such as hypertension, thyroid dysfunction and psychiatric diseases, or who were undergoing treatment with medications that could potentially increase bodyweight including amitriptyline, mirtazapine, olanzapine, quetiapine, risperidone, gabapentin, tolbutamide, pioglitazone, glimepiride, gliclazide, glyburide, glipizide, sitagliptin, and nateglinide or decrease bodyweight including metformin, acarbose, miglitol, pramlintide, liraglutide, exenatide, zonisamide, topiramate, bupropion and fluoxetine [17]. A total of 126 participants were recruited initially, and 30 students were excluded due to a lack of required participation in the intervention protocol (*n* = 10) or lack of required information (*n* = 20). Finally, a total of 96 participants including 43 students in the control group and 53 students in the intervention group were included in the final analysis.

Following the consent of the school authorities to be involved in the study, parents and students were provided with detailed information about the project and the benefits of participating, and a signed written consent form was obtained at baseline. The study was promoted to students and parents with a clear explanation of its goals, benefits and procedures. Information sessions including presentations and written materials outlined the health benefits, details of the nutritional and exercise plan, and the scientific importance of the research. Initially, all students were assessed for their socio‐demographic factors, knowledge, attitude and nutritional performance, self‐efficacy related to weight control, food intake, physical activity and anthropometric indices. Also, the students were genotyped for the rs9930506 polymorphism of the *FTO* gene.

### 2.2 Socio‐Economic Background and Lifestyle

Data on socio‐economic backgrounds were collected using a general questionnaire. Information relating to the lifestyle factors was collected through a face‐to‐face interview with the students and their mothers. The participants were evaluated regarding the status of nutritional knowledge, attitude and performance using five subscales of a validated questionnaire [18]. Using this questionnaire, the amount of nutritional knowledge of students in the field of weight‐related foods, their attitude in the field of healthy foods and snacks, and their performance in the field of nutrition were evaluated. According to the previous studies [19, 20], the scores obtained from the nutritional knowledge, attitude and performance questionnaire were classified into two categories: low knowledge (<70% of the total score) and optimal nutritional knowledge (≥70% of the total score). The level of physical activity of all participants was evaluated using a validated version of the International Physical Activity Questionnaire (IPAQ) [21].

Information on the food group intake of students during the last year was obtained by completing a 168‐item food frequency questionnaire (FFQ), which was previously validated in Iran [22] and included a list of foods with a standard serving size commonly consumed by Iranians. Portion sizes of consumed foods were converted to grams per day using household measures. Then, the Nutritionist‐IV software (First Databank Inc., Hearst Corp., San Bruno, CA) was used to analyse the intake of different types of dietary macronutrients. The daily calorie intake of the participants was calculated using the US Department of Agriculture food consumption database, which was modified for the Iranian foods [23].

### 2.3 Anthropometric Evaluations

The height of the participants was measured using a tape measure attached to the wall with an accuracy of 0.5 cm in a standing position and without shoes. Using the Bio Impedance Analyzer (BIA) scale (Omron‐BF511) [24] whose measurement accuracy has already been confirmed [24], the weight of the students was measured with an accuracy of 50 g, and after entering the age, gender and height of the student, the values related to BMI, body fat percentage and body muscle percentage were determined [25].

### 2.4 Genotyping of rs9930506 Polymorphism

A DNA extraction kit (Gene All, South Korea) was used to extract the genomic DNA from 5 mL of collected blood samples, following the manufacturer's instructions. The quality and quantity of the extracted DNA were assessed using a nanodrop (Colibri Microvolume Spectrometer; Titertek‐Berthold, Germany). The quality of the DNA was also assessed using the agarose gel electrophoresis. Amplification of the region (371 bp) surrounding the *FTO* rs9930506 polymorphism was performed using a PCR machine by the forward (5′ CAA AGG TGG GCA TAG AGA TTG 3′) and reverse (5′ ACG TGC CTA TAA AAC TGG GC 3′) primers. The PCR bands were observed and analysed by the UV Gel documentation device. The PCR products were sequenced [26] by Geneall Biotechnology Co., Seoul, Korea, and assessed using the Chromas software (Chromas DNA Sequence Analysis) in terms of the identification of the *FTO* gene rs9930506 polymorphism. Within the *FTO* gene, rs9930506 was reported to have the strongest association with BMI [14].

### 2.5 Lifestyle Intervention

Participants with overweight and obesity in the intervention group were subjected to an intense nutrition and physical activity intervention for 8 weeks. The participants in the control group continued their usual lifestyle during the study period. Based on the FFQ and anthropometric measurements, the intervention group was recommended a personalised low‐calorie and high‐protein weight loss diet according to the results of a previous study [27]. Guidance for the correct implementation of the diet was provided to each participant during face‐to‐face counselling, and written instructions were also provided for their parents. Also, three sessions of healthy lifestyle training focused on the principles of healthy nutrition and appropriate physical activity were conducted for the participants and their parents.

The sports intervention programme for participants with overweight and obesity in the intervention group was held according to the high‐intensity interval training (HIIT) method for 3 days a week, 90 min each time, for 8 weeks during the intervention. The students performed sports exercises in the playground at school on every alternate day (Saturday, Monday and Wednesday) under the supervision of a trained exercise physiologist to properly perform the sports activities and a medical practitioner to take care of possible injuries during the activity. The basis of this method was that after 10 min of relaxation, aerobics and stretching (the warm‐up), the students were divided into several groups and ran back and forth in the schoolyard at maximum speed, resting for 4–5 min between them. At the end, 5 min of stretching exercises were performed to cool down the body. These exercises became more intense as each week passed according to the protocol [28]. After completing the HIIT programme, the students played a team sport (soccer or volleyball). Furthermore, the necessary training for performing proper exercises at home was provided after each training session. To enhance adherence to the lifestyle intervention, we actively engage parents to follow their children's adherence to dietary and physical activity guidelines. Additionally, we performed periodic assessments of weight and body composition to monitor the intervention's impact and encourage sustained compliance.

### 2.6 Statistical Analysis

The normal distribution of all variables was checked using the Shapiro–Wilk test. The general linear model (GLM) repeated measure mixed ANOVA method was used to test the success rate of the weight loss intervention between the groups based on the presence of *FTO* polymorphism. Using the mixed ANOVA method, the interactions of time as within factor (baseline and after intervention) and group as between factor (intervention and control groups) were investigated. SPSS software version 23 (IBM, Chicago, IL, USA) was used for all statistical analyses, and the significance level for all analyses was considered at *p* < 0.05.

## 3 Results

The mean age of male adolescents with overweight and obesity in the intervention and control groups was 14 ± 0.99 and 14 ± 1.55 years, respectively (*p* = 0.29). No significant difference was found between the mean BMI of the intervention and control groups at baseline (26.05 vs. 26.73 km/m^2^, *p* = 0.59). The data on nutritional intake, knowledge, attitude and nutritional performance of the participants at the beginning of the study and after the intervention are presented in Table 1. At baseline, protein and carbohydrate intake in the intervention group was significantly higher than in the control group (162.34 vs. 97.15 g, *F* = 9.34, 264.4 vs. 197.15 g, *F* = 73.62, All *p* < 0.05), and fat intake was significantly lower than in the control group (135.33 vs. 203.35 g, *F* = 10.83, *p* < 0.05). After the intervention, there was a significant decrease in the intake of energy (2350 vs. 2634 kcal, *F* = 6.21), carbohydrate (255.68 vs. 286.97 g, *F* = 4.06) and fat (112.67 vs. 217.72 g, *F* = 26.65) in the intervention group compared with the control group (All *p* < 0.05). As expected, protein intake had no difference between the groups due to the recommendation of a low‐calorie, high‐protein diet for the intervention group. The level of physical activity increased after the intervention (1926.15 ± 331 vs. 3303.95 ± 703, *p* < 0.01). However, no significant difference was found regarding the increased physical activity level between the groups.

**TABLE 1:** Effect of intervention on food intake, nutritional knowledge and self‐efficacy of participants (*n* = 96).^a^

	Mean ± SD						
Parameters	Pre‐intervention		Post‐intervention		*F* values		
	Intervention (*n* = 53)	Control (*n* = 43)	Intervention (*n* = 53)	Control (*n* = 43)	Between groups	Within group	Group × Time
Energy (kcal)	2925	2550	2350	2634	0.27	3.03	6.21**
Protein (g)	162.34	97.15	158.98	106.81	9.34**	3.42	0.42
Carbohydrates (g)	264.43	197.15	255.68	286.97	73.62**	0.03	4.06*
Fat (g)	135.33	203.35	112.67	217.72	10.83*	0.42	26.65**
Total knowledge, attitude and performance	69.98	63.41	66.25	66.86	0.13	3.36	2.59
Total self‐efficacy	47.09	43.71	48.21	42.59	7.55**	0.11	0.01
Physical activity MET min/week	1926.15 ± 331	1479.16 ± 379	3303.95 ± 703	2845.79 ± 128	0.01	23.41**	2.55

^a^ With the GLM repeated measures method (df = 1) after adjusting for the confounding variables of genotype, age and anthropometric indices and significance levels of *p < 0.05 and **p < 0.01.

Regarding the effect of the intervention on the anthropometric indices, BMI in the intervention group significantly decreased (26.56 vs. 26.41 kg/m^2^, *F* = 6.58, *p* < 0.05). Also, the intervention caused a significant decrease in fat mass (27.47 vs. 26.43 kg, *F* = 10.5, *p* < 0.01). No difference was found for the other anthropometric indices measured including weight, height and body muscle percentage between the groups (Table 2).

**TABLE 2:** Effect of the intervention on anthropometric indices of participants (*n* = 96).^a^

Anthropometric indices	Mean ± SD						
	Before the intervention		After the intervention		*F* values		
	Intervention (*n* = 53)	Control (*n* = 43)	Intervention (*n* = 53)	Control (*n* = 43)	Within group	Between groups	Group × Time
Weight (kg)	74.45	74.44	75.57	76.93	0.31	3.6	0.05
Height (cm)	165.75	168.87	169.03	170.07	0.31	0.26	1.44
BMI (kg/m^2^)	26.73	26.05	26.41	26.56	0.43	0.27	6.58*
Body fat mass (%)	28.24	27.08	26.43	27.47	0.01	0.01	10.5**
Body muscle mass (%)	34.82	35.33	35.47	35.49	0.16	0.38	2.14

^**a**^ With the GLM repeated measures method (df = 1) after adjusting for the confounding variables of age, nutritional behaviour and physical activity and at the significant levels of *p < 0.05 and **p < 0.01.

The effect of the rs9930506 polymorphism of the *FTO* gene on the effects of the intervention is presented in Table 3. The genotype distribution of the study population had departed from the Hardy–Weinberg equilibrium (HWE) (AA = 28.1%, AG = 39.6%, GG = 32.3%, *p* = 0.043). The intervention had no significant effect on the anthropometric indices of GG genotype carriers compared with people with GG genotype in the control group. In other words, the intervention was not successful in improving the anthropometric indices of people with the GG genotype k. In the participants with AA/AG genotypes, the intervention group had significantly lower BMI (25.63 vs. 29.16 kg/m^2^, *F* = 6.49, *p* < 0.05) and higher body muscle (35.83 vs. 34.63 kg/m^2^, *F* = 4.43, *p* < 0.05) than the control group after the intervention (Table 4). However, these results were not significant compared with the baseline. The intervention significantly decreased BMI (−1.21 vs. 1.87 kg/m2, *F* = 4.07, *p* < 0.05) in AA/AG genotype carriers in the intervention group compared with carriers of this genotype in the control group (Table 4).

**TABLE 3:** Effect of the intervention on the anthropometric indices of people with the GG genotype (*n* = 31).^a^

Anthropometric indices	Mean ± SD						
	Before the intervention		After the intervention		*F* values		
	Intervention (*n* = 53)	Control (*n* = 43)	Intervention (*n* = 53)	Control (*n* = 43)	Within group	Between groups	Group × Time
BMI (kg/m^2^)	27.29	25.27	29.16	26.8	2.79	1.06	0.05
Body fat mass (%)	30.46	30.46	28.86	30.54	0.2	0.77	1.71
Body muscle mass (%)	33.84	34.01	34.63	34.42	0.77	4.24	1.98

^a^ With the GLM repeated measures method (df = 1) after adjusting for the confounding variables of age, nutritional behaviour and physical activity.

**TABLE 4:** Effect of the intervention on the average anthropometric indices of the AA/AG carriers (*n* = 53).^a^

Anthropometric indices	Mean ± SD						
	Before intervention		After the intervention		*F* values		
	Intervention (*n* = 53)	Control (*n* = 43)	Intervention (*n* = 53)	Control (*n* = 43)	Within group	Between groups	Group × Time
BMI (kg/m^2^)	26.84	27.29	25.63	29.16	6.49*	0.37	2.49
Body fat mass (%)	27.28	30.46	25.37	28.86	3.2	0.32	0.4
Body muscle mass (%)	35.24	33.84	35.83	34.63	4.43*	1.53	0.01

^a^ With the GLM repeated measures method (df = 1) after adjusting for the confounding variables of age, nutritional behaviour and physical activity and at the significance levels of *p* < 0.05*.

## 4 Discussion

**FIGURE 1:**
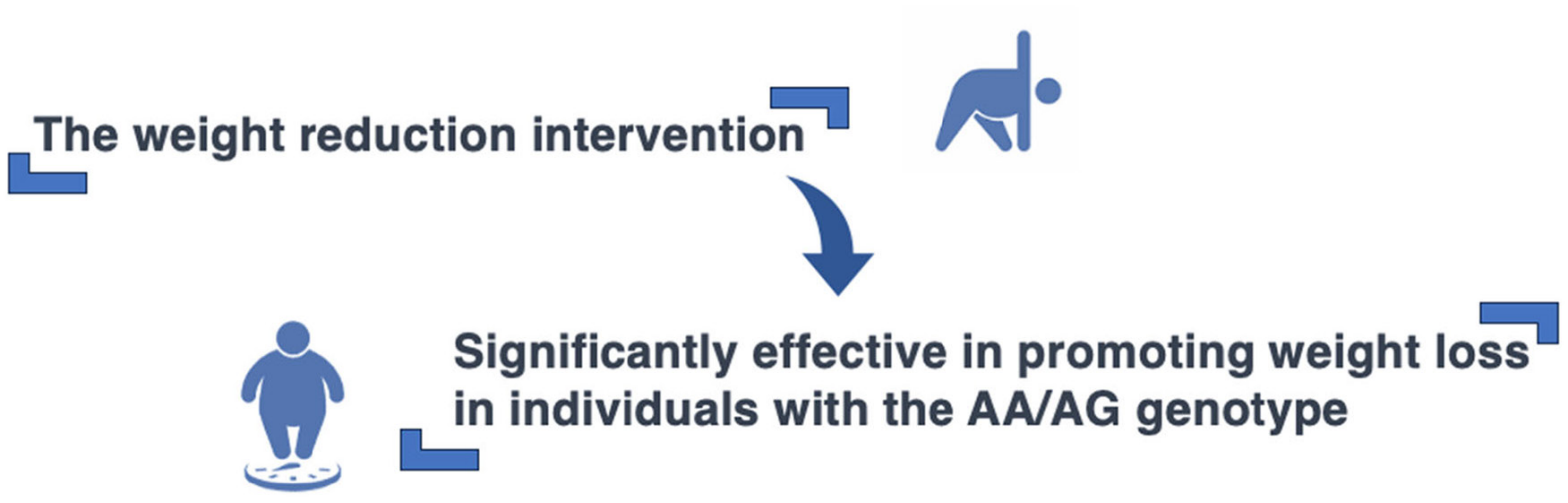
Effect of weight reduction intervention strategies in male adolescents with different *FTO* genotypes for rs9930506 polymorphism.

The results of the present study indicated that the weight reduction intervention led to a significant decrease in carbohydrate, fat and calorie intake in the intervention group compared with the control group. The intensive intervention did not have any effect on nutritional knowledge, attitude and performance in the intervention group. While there was a significant decrease in BMI and fat mass in the intervention group, no difference was evident in weight, height or body muscle percentage. The weight loss intervention did not cause any changes in anthropometric indices in the participants with the GG genotype of rs9930506 polymorphism in the intervention group compared with those in the control group. The intervention in people with AA/AG genotype was effective in weight loss compared with people with AA/AG genotype in the control group (Figure 1).

About half the world's population carries at least one of the obesity‐related risk alleles of different polymorphisms of the *FTO* gene [29]. To the best of the researcher's knowledge, no study has yet investigated the effect of rs9930506 polymorphism on weight loss interventions in male adolescents. Although most of the studies on the role of *FTO* gene genotype in obesity and body composition have been conducted on the rs9939609 polymorphism, several studies have shown that polymorphisms located in the intron of an *FTO* gene are associated with each other as haplotypes [30, 31, 32]. A meta‐analysis that examined the link between the *FTO* gene polymorphisms and obesity risk in the Chinese population found a significant association between *FTO* SNPs and increased obesity risk, with a pooled odds ratio of 1.30. This association was consistent in both children/adolescents and adults. Four specific SNPs (rs9939609, rs6499640, rs8050136 and rs1558902) showed a strong correlation with the obesity [12]. Regarding the effects of rs9939609 *FTO* polymorphism, the results showed that lifestyle factors including proper diet and physical activity may modify the influence of obesity risk alleles. Previous studies reported that the A allele of *FTO* was associated with a higher risk of obesity in individuals who follow a poor diet (e.g., a high‐fat diet) [33, 34, 35], while the effect of *FTO* risk alleles was attenuated in individuals who follow a healthy diet and regular physical activity. Findings from these studies were inconsistent with our findings, in which children carrying the homozygous genotype for *FTO* risk allele (GG) had no improvement in body weight after receiving lifestyle interventions. However, other studies focused on rs9939609 polymorphism, while in our study, rs9930506 polymorphism was investigated and different *FTO* gene polymorphisms may exert different effects. Also, the effect of *FTO* genotype on the risk of obesity in people with different diets may be different from its effect on the effect of weight loss interventions in obese people. A recent systematic review and meta‐analysis of trials on the association of the *FTO* genotype with the response to lifestyle interventions analysed 13 studies with 3980 children with overweight and obesity and found that the *FTO* risk allele had no association with changes in BMI Z‐score, BMI, waist circumference, waist‐to‐hip ratio or body fat percentage after lifestyle interventions. This result suggests that children with the *FTO* risk allele respond equally to obesity interventions compared with children without the *FTO* risk allele [36]. Furthermore, Luis et al. [37] in a nonrandomised, single‐treatment study of 44 subjects with obesity, who received a hypocaloric diet for 12 weeks, reported that body weight, BMI, waist circumference and fat mass improved without significant differences between different *FTO* genotypes ([TT] vs. [TA + AA]) of rs9939609 polymorphism. Also, Dorling et al. [38] in a recent 2‐year randomised controlled trial of 144 healthy adults without obesity, indicated that during a 2‐year caloric restriction intervention, the common *FTO* rs9939609 SNP was not associated with weight reduction, and body composition. Antonio et al. [39] in a study of 47 exercise‐trained subjects who received a hypocaloric high‐protein diet for 4 weeks reported that weight and fat loss on a low‐calorie diet were not affected by the *FTO* gene. Another meta‐analysis conducted on adults reported that individuals carrying the homozygous genotype of *FTO* risk allele may have greater weight loss than the noncarriers of the *FTO* risk allele [15]. Given that *FTO* gene expression is the highest during the growth period and decreases with age, special attention should be paid to the interactions between obesity‐related genes and diet in children and adolescents [40]. The cause of these contradictory results is not yet clear. However, the different follow‐up periods, different age groups of the studied subjects and the type of interventions may influence the results of the studies.

The underlying mechanisms of *FTO* genotype effects on anthropometric indices remain unknown. The potential role of *FTO* in regulating energy homeostasis may be effective on body weight. *FTO* expression in the hypothalamus is regulated by fasting and energy restriction [13, 41, 42, 43, 44, 45, 46]. The association between *FTO* and food intake [47, 48], habitual appetitive behaviours [49, 50] and appetite‐related hormones (ghrelin and leptin) [51, 52] has been reported in both animal and human studies. A recent study reported that the obesity‐related A allele of rs9939609 polymorphism of the *FTO* gene was associated with changes in appetite and food cravings during an intervention with hypocaloric diets [53]. In addition, the *FTO* obesity‐associated polymorphisms were associated with the expression of the homeobox gene Iroquois class homeobox protein 3 (IRX3), and the hypothalamic expression of IRX3 gene is reported to be associated with body composition and calorie intake [54].

The main strength of this study was the interventional design in adolescents with overweight and obesity, and the results were obtained from a relatively homogeneous population. However, the present study has several limitations that warrant consideration. First, only one school was assigned to each condition, and differences between schools may influence the outcomes. Second, it is important to note that the study exclusively involved male adolescents, which may limit the generalisability of the findings to other populations. Third, only one SNP of the *FTO* gene was analysed. Given that participants might carry different SNPs in this gene or in other genes related to obesity, this could potentially affect the observed phenotype. Fourth, the duration of the study was relatively short that could influence the outcomes. Lastly, as the study focused on adolescents with overweight and obesity, the results do not provide insights into the role of the *FTO* gene in preventing obesity among adolescents.

## 5 Conclusion

In summary, the results of this study indicated that the weight reduction intervention in individuals with the AA/AG genotype of the *FTO* gene has been significantly effective in weight loss. The intervention had no significant effect on anthropometric indices of adolescents with the GG genotype of the *FTO* rs9930506 polymorphism. Future similar studies may help to fill a research gap by exploring how genetic variations influence the outcomes of lifestyle interventions, addressing inconsistencies in previous findings and contributing to tailored obesity treatment strategies based on genetic profiles. More randomised controlled intervention trials in adolescents with overweight and obesity are thus needed to investigate the role of the *FTO* genotype in the success of obesity interventions. If the results of this study are confirmed in future studies, the information related to the gene sequence can be used to provide personalised diets to treat obesity.

## Author Contributions

**Zahra Roumi:** Resources. **Zahra Salimi:** Data collection. **Zahra Mahmoudi:** Data collection. **Khadijeh Abbasi Mobarakeh:** Data collection. **Maryam Ladaninezhad:** Data collection. **Mobina Zeinalabedini:** Data collection. **Mohammad Keshavarz Mohammadian:** Data collection. **Ali Shamsi‐Goushki:** Validation. **Zahra Saeedirad:** Data collection. **Bojlul Bahar:** Design the study. **Sara Khoshdooz:** Data analyses. **Naser Kalantari:** Design the study. **Ghasem Azizi Tabesh:** Laboratory works. **Saeid Doaei:** Data analyses. **Maryam Gholamalizadeh:** Design the study.

## Ethics Statement

This study was approved by the Ethical Review Board at Shahid‐Beheshti University of Medical Sciences, Tehran, Iran (Code Ir.sbmu.nnftri.rec. 1397.1249).

## Consent

Institutional consent forms were used in this study. All participants were assured that the collected information would remain confidential and that they could withdraw from the study at any time. A written consent form was obtained from all participants and child parents at baseline (Trial Registration Number: IRCTID: IRCT2016020925699N2).

## Conflicts of Interest

The authors declare no conflicts of interest.
